# Optimization and Standardization of Stable De-Epidermized Dermis (DED) Models for Functional Evaluation of Cutaneous Cell Therapies

**DOI:** 10.3390/bioengineering11121297

**Published:** 2024-12-20

**Authors:** Xi Chen, Corinne Scaletta, Zhifeng Liao, Alexis Laurent, Lee Ann Applegate, Nathalie Hirt-Burri

**Affiliations:** 1 Plastic, Reconstructive and Hand Surgery Service, Lausanne University Hospital, University of Lausanne, CH-1011 Lausanne, Switzerland; xi.chen.1@unil.ch (X.C.); corinne.scaletta@chuv.ch (C.S.); zhifeng.liao@unil.ch (Z.L.); alexis.laurent@unil.ch (A.L.); 2 Manufacturing Department, LAM Biotechnologies SA, CH-1066 Epalinges, Switzerland; 3 Lausanne Burn Center, Lausanne University Hospital, University of Lausanne, CH-1011 Lausanne, Switzerland; 4 Center for Applied Biotechnology and Molecular Medicine, University of Zurich, CH-8057 Zurich, Switzerland; 5 Oxford OSCAR Suzhou Center, Oxford University, Suzhou 215123, China

**Keywords:** air-liquid interface, burn wound exudates, cutaneous cell therapies, de-epidermized dermis (DED), extracellular matrix, HaCaT, in situ model, keratinocytes, serum-free media, tissue regeneration, tissue engineering

## Abstract

The human skin is a remarkable organ capable of extensive regeneration, especially after severe injuries such as burns and related wounds. The de-epidermized dermis (DED) model has become a valuable in vitro tool for skin regeneration studies, particularly for testing the mechanism of action and the efficacy of clinical cutaneous cell therapies. To further improve the quality and robustness of these applications, our study focused on optimizing and standardizing DED tissue preparation and storage, enhancing its effectiveness for clinical testing. Therefore, we optimized the air-liquid interfacial culture medium composition by simplifying the historical formulation without compromising keratinocyte (therapeutic cell model) viability or proliferation. Furthermore, we investigated the impacts of adding burn wound exudates in the model by focusing on cell behavior for enhanced translational significance. The results revealed notable differences in keratinocyte adhesion and proliferation between burn wound exudates collected at the early stages and late stages of acute patient treatment, providing new information on a possible therapeutic window to apply cell therapies on burn patients. Generally, this study reported a robust method for the preclinical in vitro assessment of keratinocyte-based cutaneous cell therapies using DED models. Overall, the study underscored the importance of using in vitro models with enhanced translational relevance to better predict the clinical effects of cutaneous cell therapies in burn patient populations.

## 1. Introduction

De-epidermized dermis (DED) is a cutaneous tissue preparation that is widely used in medical and research settings, particularly in wound healing studies [1]. DED preparation involves the removal of the outer epidermal layer of the skin, leaving behind a scaffold of extracellular matrix (ECM) proteins [2]. Of note, this model is valuable for culturing cells at the air-liquid interface, offering insights into cellular attachment, proliferation, and differentiation, mimicking physiological conditions [3,4,5]. Additionally, the DED model (i.e., lacking keratinocytes and fibroblasts) can be used to simulate burn wounds, making it highly useful for evaluating topical cellular therapeutic approaches [1]. There is currently a need to work with biological assays to screen and determine the mechanistic actions of cutaneous cellular therapies. The assays proposed herein, based on DED and in vitro organo-culture systems, should help to answer pertinent questions on burn wound management and mechanisms of action for improved rapidity, safety, and efficacy before moving on to animal models.

Historically, the concept of DED emerged from research in regenerative medicine, but more importantly, for direct use of the prepared dermal tissues for clinical application to help cover large surface wounds [6]. Early experiments using epidermis-free dermis for skin tissue regeneration date back to the last century [2]. Researchers initially utilized materials such as postmortem pig skin, as reported by Freemann et al. in 1976, and later with human cadaver skin by Wainwright in 1995 or Boelsma in 1999 [5,6,7,8]. Over time, various methods were explored to prepare DED, including chemical and enzymatic techniques for gentle epidermal layer removal while preserving dermal structures [4,9]. To date, the method involving overnight incubation with 1 M NaCl, pioneered by MacNeil’s group [4], gained prominence for its effectiveness in epidermis removal and was subsequently adopted by other research teams [10]. The clinical use of DED is still relevant today to provide early cover for severe burn patients and to avoid extensive fluid loss. However, this is only a temporary treatment while waiting for therapeutic cellular formulations and skin grafting for patient care.

Thereafter, DED was also adapted to develop an experimental model with the air-liquid interface to mimic human skin physiological conditions. For this model, keratinocytes are seeded on DED and overlaid on a grid in contact with both air and liquid medium, leaving the upper layer exposed only to air. Indeed, human skin is composed of multiple layers of keratinocytes attached to the underlying dermis, with the inner layers containing proliferating cells in direct contact with the dermis. The outer layers are made up of differentiated cells that form the surface of the skin. These cells are constantly exposed to air, with nutrients supplied from the dermis via diffusion from blood vessels, which is comparable to the DED air-liquid interface model [2,11]. Therefore, this in vitro organo-culture system can be exploited to study new formulations with different cell types, ratios of cells, and efficient delivery of cells to provide better tissue repair.

Keratinocyte proliferation medium, pioneered by Rheinwald and Green in the 1970s [12], allows the in vitro culture and expansion of keratinocytes on a 3T3 fetal mouse fibroblast feeder layer, which is also employed in the context of DED to nourish the seeded keratinocytes [13,14]. This medium has a complex formulation including DMEM, Ham’s F12, fetal bovine serum (FBS), insulin, epidermal growth factor (EGF), hydrocortisone, cholera toxin, and antibiotics. Over the years, researchers have modified the formulation by adding or removing specific components to suit their experimental needs [13,14,15,16]. In our experiments, we aimed to simplify the medium composition by reducing specific components without compromising its effectiveness with regard to the DED model, cell adhesion, and cell migration. We retained the basal medium containing DMEM and Ham’s F12, which provided amino acids and vitamins, along with FBS, which is crucial for maintaining physiological balance and promoting cellular proliferation [15,16]. Furthermore, we evaluated the removal of cholera toxin, hydrocortisone, insulin, and EGF as others have also looked at certain specific elements such as cholera toxin [17].

For burn wound models, the use of human burn wound exudate in association with DED could be beneficial in studying cellular mechanisms of repair and regeneration. Exudates are fluids produced by the body in response to tissue damage. To date, the most significant known utility of exudates is their use in burn wound analysis. In the work by Widgerow et al. [18], an analysis of 29 relevant articles from PubMed highlighted the importance of burn wound exudates in understanding the tissular environment of burns. Indeed, this element provides insights into local tissue damage and wound depth through analysis of cellular components, which aid in wound diagnosis [18,19]. Furthermore, these fluids contain a multitude of components, including cytokines, growth factors, proteins, and cellular debris, which are crucial for wound healing and regeneration [20,21]. It is, therefore, interesting to study this fluid as a potential tool for improving cellular wound healing potential, although only a few scientific studies have examined its proliferative, migratory, or adhesion effects on cutaneous cells [21]. 

In this work, we aimed to further standardize the DED production method, providing a detailed and simplified description of the de-epidermization processes for small to mid-size batch preparation and long-term preservation to ensure uniform off-the-fridge usability. With the DED model combined with an air–liquid interface, the composition of Green’s culture medium was examined in detail to propose alternative formulations and to find a simplified medium without compromising its effectiveness on cellular interactions. We also aimed to elucidate the impact of burn wound exudates on cellular attachment in an in vitro DED air-liquid interface model. To this end, exudates collected at different time points from burn patients were used to study their effects on keratinocyte behavior on the DED interface. The HaCaT cell line was used in our experiments to validate the DED model, perform DED culture medium optimization, and evaluate the impact of adding burn wound exudates. The HaCaT source is a well-established, immortalized human keratinocyte cell line derived from normal human skin that spontaneously transformed [22]. HaCaT cells provide a reliable model for reconstructing a human skin equivalent [5]. Generally, the primary hypothesis of the work was that simplified keratinocyte proliferation media can be effectively used in DED models, and the secondary hypothesis was that the addition of burn wound exudate to the model significantly impacts the behavior of seeded keratinocytes. Overall, the novelty and significance of the presented work was the use of optimized methods for DED model setup and the use of patient burn wound exudates in order to obtain an in vitro model with high translational relevance.

## 2. Materials and Methods

### 2.1. Ethical Compliance for Skin Tissue and Exudate Procurement

Skin samples were obtained from the DAL biobank [Biobank DAL, Biobank of the Musculoskeletal Medicine Department of the Lausanne University Hospital, CHUV, (BB_029_DAL)] after patient informed and written consent. Anonymized human skin tissue derived from “waste” tissue of abdominoplasties was placed in a sterile container in the operating arena and was transferred to the laboratory for processing.

Exudates from burn wounds were collected at the Burn Care Unit of the University Hospital of Lausanne (CHUV) after approval by the Ethics Committee of the State of Vaud, Lausanne, Switzerland (Ethics protocol #488/13, 2013) and integrated into the DAL Biobank (BB-029_DAL), which was also evaluated by the State Ethics Committee. Exudates were placed in a sterile container in the operating arena and were transferred to the laboratory for processing.

### 2.2. DED Tissue Preparation Method

**Figure 1:**
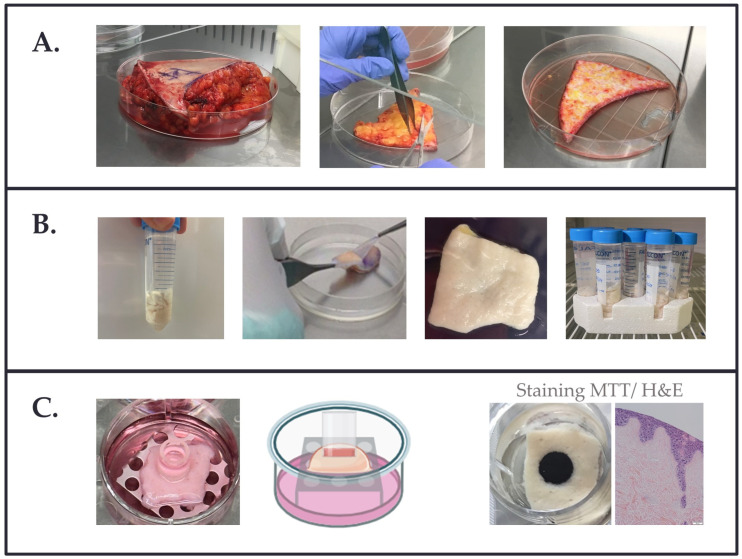
(**A**) Abdominal tissue was treated to remove the adipose tissue with surgical scissors and was cut into strips. (**B**) Tissue strips were transferred to 50 mL Falcon tubes, which were filled with NaCl 1 M. After a 24 h/37 °C incubation phase, the epidermis was separated from the dermis with forceps. Each tissue strip was then cut into individual samples of ~1.5 cm^2^ and placed into Falcon tubes with 1× PBS + 1% P/S. The solution was changed 2–3 times before processing for long-term storage. (**C**) Description of the DED model with the air–liquid interface. A sterile perforated metal support was positioned at the bottom of a 6-well plate. The DED was first incubated in complete culture medium for at least 2 h and carefully transferred onto the support, papillary side up. Selected culture media (~4 mL/well) were added to ensure nutrient perfusion. A 6 mm glass insert was gently placed in the center of the DED, allowing for 100–200 µL of cell seeding. Constructs (i.e., DED + cells) were maintained for 4 days at 37 °C, 5% CO_2_. Then, the glass inserts were removed, and the constructs were incubated for 7 more days. Cellular presence and surface repartition were assessed by MTT staining of the whole construct and by H&E staining of 7 µm histological sections. DED, de-epidermized dermis; PBS, phosphate-buffered saline; MTT, 3-(4,5-dimethylthiazol-2-yl) 2,5-diphenyltetrazolium bromide.

The ex vivo DED model was initially established as previously described [23]. All the procedures were performed under a laminar hood in a sterile environment. Briefly, abdominal tissue (i.e., ranging from 10–25 cm in length and ~10 cm in height) was processed within 1–4 h following transfer from the operating arena (Figure 1A). Large tissue donations from abdominoplasties enabled the preparation of various lot sizes of tissue samples (i.e., 1.0–1.5 cm^2^) destined for multiple experiments. Quality controls of the produced DED were performed using H&E staining of histological sections for each lot. Briefly, the tissue was first treated with surgical scissors to remove adipose tissue fragments. The tissue was then cut into smaller portions of approximately 10 × 10–15 cm. These tissues were transferred to sterile 15 cm Petri dishes, and several washes were performed with phosphate-buffered saline 1× (PBS; Bichsel, Unterseen, Switzerland) with 1% penicillin-streptomycin (P/S; Life Technologies, Paisley, UK). Further removal of the remaining adipose tissue was accomplished with dissecting scissors. Lengths of tissue of 1.5 × 10–15 cm were prepared for the de-epidermalization procedure. Firstly, the tissue was covered with PBS + 1% P/S for 15 min. Tissue strips (i.e., up to 5 per tube) were transferred to 50 mL Falcon tubes filled with ~30–40 mL sterile-filtered NaCl 1 M (Bichsel, Unterseen, Switzerland; Figure 1B). The tissues were incubated at 37 °C overnight with moderate orbital shaking. Following 24 h of incubation, the tissues were transferred to 10 cm Petri dishes, and the epidermis was separated from the dermis with forceps. The tissues were washed two times with sterile PBS and transferred to new 50 mL Falcon tubes. PBS + 1% P/S (i.e., ~30–40 mL) was added to each tube, and the tissues were incubated for 24 h at 4° C with gentle orbital shaking. Then, the PBS + 1% P/S was changed once, and another incubation was accomplished before the tissue was processed and conditioned for storage at 4 °C, −20 °C, or −80 °C. For this phase, each tissue strip was cut into individual samples (~1.5 cm^2^), and 12 samples/tube were conditioned into 50 mL Falcon tubes with PBS + 1% P/S (~30–40 mL/tube) for 4 °C storage until use. For the preparation of frozen DED lots, the tissues were frozen in dry form (i.e., without the addition of a freezing medium or buffer) in tissue culture plates covered with parafilm.

As regards the mitigation of donor-to-donor variability, the possibility of preparing several tissue samples from the same large tissue donation enabled the conducting of an experimental series with a single donor. Performing comparative quality controls between DED lots then enabled the exclusion of extensive structural variability. Of note, the possibility for harvesting tissue from the same donor anatomical sites has also helped to standardize DED tissue preparation further. Moreover, the standardized method of preparation of the DED was then assessed with H&E staining, and histological structural controls for quality between lots could be confirmed. Finally, the intensive salt treatments that were applied to the processed tissues were considered to inhibit antigen-type reactions, as all of the cellular structures were eliminated.

### 2.3. Preparation of the Air–Liquid DED Construct with HaCaT Keratinocytes

The ability of HaCaT cells to form a multilayered epithelium when cultured at the air-liquid interface makes them useful for human skin research and provides a basis for a standardized skin model. The HaCaT keratinocyte cell line was maintained in a complete culture medium composed of DMEM (Thermo Fisher Scientific, Waltham, MA, USA), with 10% FBS (Merck, Darmstadt, Germany), and 1% L-glutamine (Thermo Fisher Scientific, Waltham, MA, USA). To prepare the DED construct, a perforated metal support measuring 2 × 2 × 0.5 cm was positioned at the bottom of a 6-well plate (Figure 1C). The needed amount of 1.5 cm^2^ DED (i.e., stored within 50 mL Falcon tubes) was rinsed with PBS and incubated in complete culture medium for at least 2 h. Then, the DED was carefully transferred onto the sterile support (i.e., papillary side up), and ~4 mL of medium were added. A glass insert was gently placed in the center of the DED using forceps to aid in cell containment and localization. Volumes of 100 µL culture medium containing 20,000 HaCaT cells were seeded into the glass inserts on top of the DED air–liquid interface model. Specifically, the cells with culture medium were placed on top of the DED inside an insert to ensure cell localization. The cells were cultured for 4 days at 37 °C and 5% CO_2_, after which the inserts were removed, the DED culture medium was changed, and constructs (i.e., DED + cells) were maintained for one week. Additional medium exchanges were accomplished after 3 to 4 days.

### 2.4. Media Optimization for the Air-Liquid Interface of DED Constructs

Green’s medium was developed for supporting keratinocyte cultures on 3T3 embryonic mouse fibroblast feeder layers (i.e., 3T3 cells irradiated with gamma rays or treated with mitomycin C) [12]. This medium has been previously used by other groups to maintain the cells seeded on the air–liquid DED constructs. The Green’s medium composition used in this work was a slightly modified version from the one described by Rheinwald and Green [12] (i.e., hereafter denominated as mGreen’s medium) and included DMEM and Ham’s F12 (Merck, Darmstadt, Germany) with a 3:1 proportion, 0.14 nM cholera toxin (Lubio Science, Zurich, Switzerland), 332.9 ng/mL hydrocortisone (Pfizer, New York, NY, USA), 8.3 ng/mL EGF (Merck, Darmstadt, Germany), 832.2 µM L-glutamine (Thermo Fisher Scientific, Waltham, MA, USA), 0.12 U/mL insulin (Novo Nordisk Pharma, Bagsværd, Danemark), and 10% FBS (Merck, Darmstadt, Germany). Firstly, different media were tested for the cell seeding step and for the DED maintenance phase (i.e., complete medium, mGreen’s medium, and the serum-free medium CnT-PR [CellnTec, Bern, Switzerland]) to find the best combination in terms of cellular behavior. Then, simplified formulations of the mGreen’s medium were tested to assess if a simpler version could be used in a controlled assay without impacting cellular behavior. The tested media compositions are shown in Table 1, ranging from all components used in the parent formulation (i.e., mGreen’s medium) to those with less complexity (i.e., complete medium).

### 2.5. MTT Staining of DED Constructs

To assess cell viability on the DED constructs, a 3-(4,5-dimethylthiazol-2-yl) 2,5-diphenyltetrazolium bromide conversion assay (MTT; Thermo Fisher Scientific, Waltham, MA, USA) was performed in parallel on DED samples. To do so, DED constructs were transferred into 12-well plates, and 1–2 mL of 0.5 mg/mL MTT in PBS (Bichsel, Unterseen, Switzerland) were added, followed by a 2 h incubation period at 37 °C. Samples were washed twice with PBS, and imaging was performed macroscopically using an iPhone 15 (Apple, Cupertino, CA, USA; Figure 1C). Metabolically active cells were stained in purple. Stained DED samples were fixed in formalin for histology and subsequent H&E staining of 7 µm sections. The diameter of viable MTT staining (i.e., in purple) was measured using photographic images, and the intensity of the staining was analyzed using Image J (v. 1.52t; NIH, Bethesda, MD, USA). Images were imported using the “File > Open” command, and the region of interest was defined using the ellipse selection tool. The color intensity within the selected region was measured with the “Analyze > Measure” function. 

### 2.6. Histology Processing of the DED Constructs

To perform histology of the DED constructs, they were sectioned into 0.5 × 0.5 cm^2^ pieces and placed into cassettes before being submerged in a container with 4% *m/v* formaldehyde (Sigma Aldrich, Buchs, Switzerland) for fixation. The tissues were incubated overnight, washed with 1× PBS, and stored at 4 °C in 70% ethanol until paraffin embedding. Then, the DED samples were embedded in paraffin wax blocks (Sigma Aldrich, Buchs, Switzerland) and were sectioned using a microtome to a thickness of 7 μm before being placed on glass slides. Hematoxylin & eosine (H&E; Sigma Aldrich, Buchs, Switzerland) were used to stain the slides. This was accomplished by deparaffinizing the embedded sample sections in xylene (Sigma Aldrich, Buchs, Switzerland; i.e., twice for 10 min). They were then passed through 100% ethanol (Chemie Brunschwig, Basel, Switzerland; i.e., twice for 10 min), 94% ethanol (i.e., once for 10 min), and 70% ethanol (i.e., once for 10 min). After staining of nuclei and basophilic substrates for 15 min in hematoxylin, the samples were quickly submerged 1–2 times in an acidified alcohol solution. Finally, after rinsing and incubating with water for 30 min, acidophilic substrates were stained with erythrosine. Histological samples underwent a dehydration process (i.e., ascending alcohol series) and xylene clarification before they could be mounted using Eukitt mounting agents (Biosystems, Bobingen, Germany) and a cover glass. Imaging was performed with an Olympus IX81 microscope (Olympus Corporation, Tokyo, Japan; Figure 1C). The histology readout was described as the level of epidermal definition, which includes the keratinocyte adhesion and invagination within the dermis, where + represents “rare cells and no invagination”, ++ represents “low cellular layering and low invagination”, and +++ represents “normal skin-like epidermal cell layering and significant invagination”.

### 2.7. Immunohistochemistry of DED Constructs

DED constructs with no MTT staining were fixed and used for immunohistochemistry experiments. Immunohistochemistry was employed to identify selected proteins in the different experiments with the DED constructs. Several key proteins were tested, including laminin 1, collagen IV, and cytokeratin 14 (K14). Laminin 1, primarily located in the skin basement membrane, plays a crucial role in cellular adhesion and migration [24]. Collagen IV, also found in the skin basement membrane, contributes to the structure and stability of this membrane [24,25]. K14 is a marker of keratinocyte differentiation, present in epithelial layers, indicating a good level of cellular maturation and differentiation on the DED [5,26,27]. The results from each experimental condition were compared to the normal skin structures observed in vivo, which may provide the essential basis for tissue engineering applications developed for burn treatments.

The embedded sample sections were deparaffinized by immersing them twice in xylene for 10 min each, followed by sequential immersion in 100% ethanol (i.e., twice for 10 min), 94% ethanol (i.e., once for 10 min), and 70% ethanol (i.e., once for 10 min). The sections were then washed and incubated with 0.25% PBST for 10 min to allow permeabilization. After washing, the sections were treated with proteinase K (PanReac AppliChem, Darmstadt, Germany) for 15 min at 37 °C for epitope unmasking. Endogenous peroxidase activity was blocked using Bloxall (SP-6100-100; Vector Laboratories, Newark, CA, USA) for 10 min, followed by a 1 h incubation with 2.5% horse serum (ref. 30022; Vector Laboratories) to block non-specific binding. The slides were then incubated overnight at 4 °C with the primary antibody corresponding to the different tested proteins (Table 2).

The next day, after washing, the appropriate secondary antibody (i.e., either ImmPress horse anti-rabbit [MP-7401; Vector Laboratories] or horse anti-mouse [MP-7402; Vector Laboratories]) was applied, and the slides were incubated for 1 h. Staining was visualized using ImmPact DAB (SK-4105; Vector Laboratories) under the microscope for less than 2 min, depending on the coloration intensity. Following immunohistochemical staining, the slides were stained with hematoxylin and then subjected to a dehydration process using an ascending series of alcohols and xylene clarification before being mounted with Eukitt and glass coverslips. Negative controls were prepared by omitting the primary antibodies. The scanner microscope Olympus VS200 (Olympus Corporation, Tokyo, Japan) was used to perform imaging.

### 2.8. Burn Wound Exudate Collection and Use

As described previously [28], the exudates from superficial burns of the second or deeper degree were collected from burn patients in our burn center (Lausanne University Hospital, Lausanne, Switzerland). Sample collection began when the patient was first admitted to the burn center, and a negative pressure dressing was used to collect fluid into a reservoir bottle. During the first week following trauma, exudate samples were taken twice per day (i.e., in the morning and the evening) by changing the reservoir bottle. As soon as the wound site was grafted or the exudation naturally stopped, sample collection was stopped. Samples obtained were kept at −80 °C before use. In the reported experiments, exudates from three patients, collected at different time points, were analyzed: (i) “Early exudate” from early-stage exudates collected shortly after the injury occurred (i.e., pooled samples from day 1 and day 2), and (ii) “Late exudate” from exudates collected several days post-injury (i.e., pooled samples from day 4 to day 6). For use in the DED model with the air-liquid interface, the exudates were used in undiluted form in place of the culture medium [28]. These two distinct phases were taken from global data from burn patients included in the Biobank, and major changes were found in trace elements from 1–2 days to the later 4–6 days of exudation [29].

### 2.9. Impact of Early and Late Collection Exudates on Adult Primary Fibroblast Survival

Two primary fibroblast cell types from burn patients were used from the DAL Biobank. Cells were seeded at 6000 cells/well in 24-well plates (Costar, Corning, NY USA) in a culture medium composed of DMEM, with 10% FBS, and 1% glutamine. When the cells reached 80% confluency, the medium was removed and replaced with burn wound exudates from early (i.e., 1–2 days post burn) and late (i.e., 4–6 days post burn) collection for 48 h. The exudate was then removed, cells were washed with PBS, and a cell titer assay was performed according to the manufacturer’s recommendations (Cell titer; Promega, Madison, WI, USA). Results were expressed as a percentage of control cells (i.e., grown continuously in culture medium) ± SD. Pictures of cell cultures were taken at the end of the experimentation at 48 h.

### 2.10. Statistical Analysis and Data Presentation

The calculations (Student’s *t*-test) and data presentation were performed using Microsoft Excel v2411 and Microsoft PowerPoint v2411 (Microsoft Corporation, Redmond, WA, USA).

## 3. Results

### 3.1. Evaluation of Media for Cell Seeding of DED Constructs and Air-Liquid Interface Culture

To evaluate the optimal combination of medium for the HaCaT cell seeding on top of the DED and the air–liquid interface culture medium, we have tested different combinations as follows: (i) complete culture medium and CnT-PR serum-free medium for the cell seeding and (ii) mGreen’s medium and CnT-PR serum-free medium for the DED construct culture.

**Figure 2:**
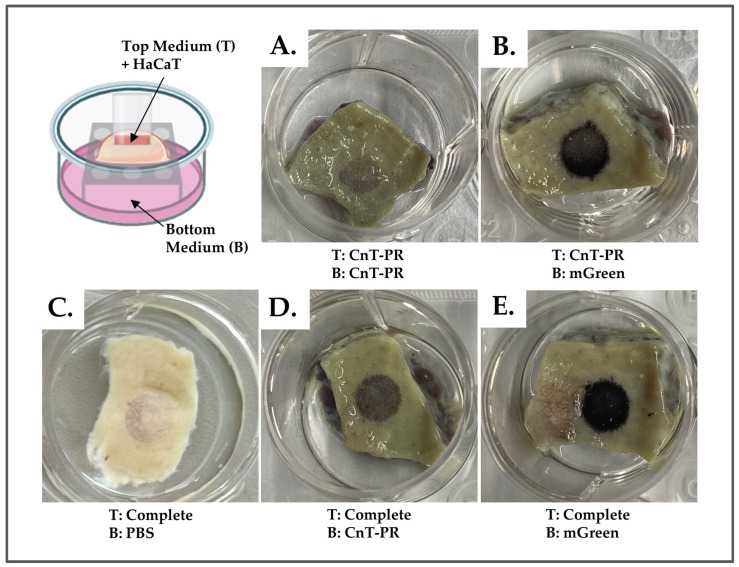
Macroscopic images of an MTT assay for whole DED tissue samples, where media combinations were compared. The top medium (T) was used to fill the glass insert positioned on the DED. The bottom medium (B) was the DED culture medium. (**A**) Serum-free medium on both top and bottom. (**B**) mGreen’s medium on the bottom, and serum-free medium on the top. (**C**) Control sample with PBS on the bottom and complete medium on the top. (**D**) Serum-free medium is on the bottom, with complete medium on the top. (**E**) mGreen’s medium on the bottom, with complete medium on the top. DED, de-epidermized dermis; MTT, 3-(4,5-dimethylthiazol-2-yl) 2,5-diphenyltetrazolium bromide.

**Figure 3:**
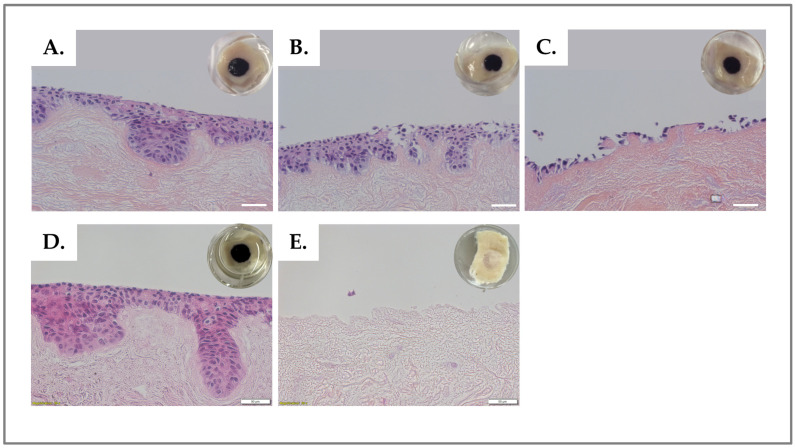
The effect of the conservation temperature on DED prior to recellularization with HaCaT cells (i.e., with mGreen’s medium for the air–liquid culture) was evaluated by MTT staining and histological analysis of H&E tissue sections. The MTT assay stains viable and metabolically active keratinocytes and reveals the cell distribution on the DED macroscopically. Histological H&E staining allows for morphological analysis of the stratified epidermal layer with respect to cellular adhesion and migration within the dermal structure. The figure shows DED cross-sections embedded in paraffin, cut at 7 µm, and stained with H&E. Storage was performed at (**A**) 4 °C for six weeks; (**B**) −20 °C for six weeks; (**C**) −80 °C for six weeks; (**D**) 4 °C for five years. (**E**) Control group with PBS alone and no nutritive media, stored at 4 °C for six weeks. Upper right corners = MTT staining in macroscopic imaging. Scale bars = 50 µm. DED, de-epidermized dermis; MTT, 3-(4,5-dimethylthiazol-2-yl) 2,5-diphenyltetrazolium bromide; PBS, phosphate-buffered saline.

Figure 2 and Figure 3 macroscopically show the MTT staining of the different DED constructs. The staining revealed distinct patterns corresponding to cellular viability and the degree of cell adhesion to the DED. The intensity of the stain color was proportional to the number of adherent cells. However, from the macroscopic images, it was not possible to evaluate the depth of cellular colonization, as could be seen in the histological sections. It was observed that mGreen’s medium for the bottom medium (B) exhibited more pronounced staining (Figure 3B,E) compared to the CnT-PR serum-free medium (Figure 2A,D). Additionally, the top medium (T) for HaCaT cell seeding with complete medium (Figure 2D,E) demonstrated enhanced MTT stain intensity compared to CnT-PR medium (Figure 2A,B). When there was no medium to feed the DED, as seen in Figure 2C, the cells were unable to survive and develop proper adhesion to the DED. Altogether, the best media combination to prepare the DED model for testing the construct with HaCaT cells was to seed the cells in a complete medium and to culture the construct in mGreen’s medium. These results are consistent with the conditions described by most research groups.

### 3.2. Stability Studies: Storage Conditions of DED

After decellularization, DED samples were stored for six weeks either at 4 °C in 1× PBS + 1% P/S or with no liquid at −20 °C and −80 °C. After gentle thawing, DED constructs were seeded with HaCaT cells in complete medium and mGreen’s medium for the air-liquid culture phase. The experiment was performed with two different DED lots. The results of H&E staining revealed better HaCaT proliferation and deeper dermal penetration for the DED stored at 4 °C compared to lower temperatures (Figure 2A–C). Notably, DED can be stored at 4 °C for up to five years (Figure 2D) without affecting recellularization, thus allowing for the preparation of large tissue lots that may be banked with uniformity. The 4 °C storage protocol was thus used for all experiments presented herein.

### 3.3. Culture Media Composition Screening

**Figure 4:**
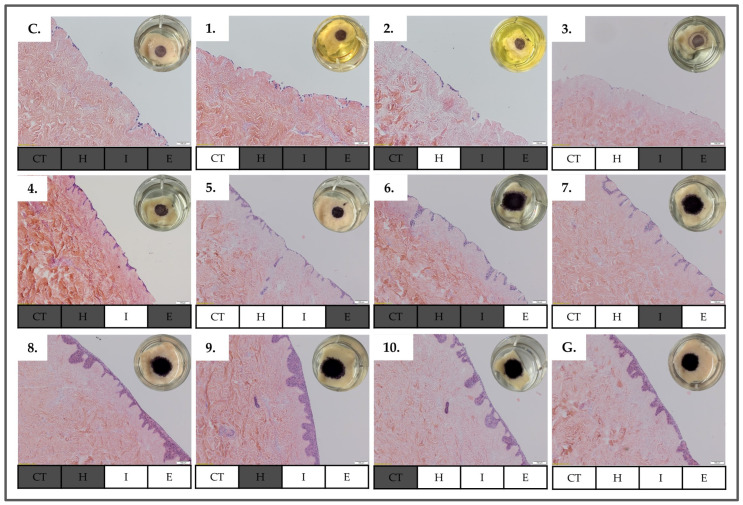
Results of culture media composition screening. (**C**) Control group. (**1**–**10**) Medium formula N.1–N.10. (**G**) mGreen’s medium. DED was evaluated macroscopically by MTT and histological analysis of H&E tissue sections. The MTT assay stains viable and metabolically active keratinocytes and reveals the cell distribution on the DED macroscopically. Histological H&E staining allows for morphological analysis of the stratified epidermal layer with respect to cellular adhesion and migration within the dermal structure. H&E results and MTT results (i.e., upper right corners). The absence of specific medium components is represented by dark gray highlighting. Scale bars = 100 µm. C, complete medium; CT, cholera toxin; E, EGF; EGF, epidermal growth factor; G, mGreen’s medium; H, hydrocortisone; I, insulin; MTT, 3-(4,5-dimethylthiazol-2-yl) 2,5-diphenyltetrazolium bromide.

We have shown that mGreen’s medium was the medium that performed with the best results for maintaining the DED constructs (Figure 2). As this medium is complex, we aimed to evaluate the possibility of simplifying the formulation. To achieve this goal, we have tested each of its major components separately or in different combinations to assess if it is possible to obtain the same results as for the mGreen’s medium. MTT-stained DED construct images reported in Figure 4 show a range of different staining intensities among the tested conditions, attesting that the different components of mGreen’s medium impact the cellular viability of HaCaT cells to different degrees. When DED constructs are cultured in a complete medium or in a medium with cholera toxin, hydrocortisone, or both, the MTT staining showed the same intensity as that of the complete medium. However, when insulin was added, with (Figure 4, #5) or without (Figure 4, #4) cholera toxin and hydrocortisone, a small increase in staining intensity was observed. Similarly, when EGF replaced insulin (Figure 4, #6–7), there was a clear enhancement in staining intensity compared to all other formulations (Figure 4, #C,1–5). Interestingly, conditions corresponding to images 8 to 10, indicating the presence of both insulin and EGF (i.e., with or without cholera toxin or hydrocortisone), showed similar staining intensity and proliferation than with the one observed for the “Green’s medium” (Figure 4, #G) containing all the mentioned components.

The results of the H&E staining of DED construct histological sections (Figure 4) supported those of the macroscopically illustrated MTT assay. Conditions corresponding to images 1 to 3 exhibited almost no cells attached to the surface of the DED, and no invagination of keratinocytes was formed like that observed with the complete medium (Figure 4, #C). With the addition of insulin (Figure 4, #4–5), a slight increase in cell attachment and keratinocyte invagination was noted. The substitution of insulin with EGF (Figure 4, #6–7) resulted in an increase of attached cells and cell invagination on the DED construct. The best results were obtained with media compositions containing both insulin and EGF, which demonstrated high cellular proliferation and invagination comparable to the use of mGreen’s medium for the air–liquid interface (Figure 4, #8–10,G). Notably, images 6 to 7, representing the addition of EGF but lacking insulin, showed a significant reduction of keratinocyte invagination compared to samples with both EGF and insulin (Figure 4, #8–10,G), a discrepancy which was not evident in the macroscopic MTT assay results alone.

Table 3 summarizes the results, classifying the color intensity and macroscopic diameter of the MTT stain, as well as the epidermal definition of keratinocyte presence and level of invagination with the H&E stain from low to high.

**Figure 5:**
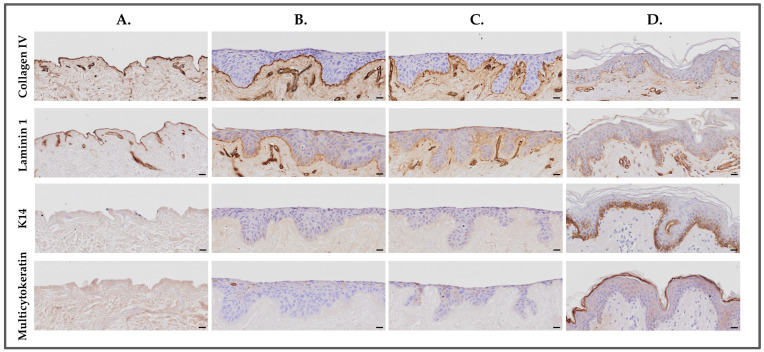
Immunohistochemical analysis of collagen IV, laminin 1, and K14 on DED constructs maintained in mGreen’s medium and the new simplified medium N°8. (**A**) Control group. (**B**) New simplified medium N°8. (**C**) mGreen’s medium. (**D**) Human skin control group. Scale bars = 20 µm. DED, de-epidermized dermis.

Immunohistological staining was performed on the DED constructs cultured with mGreen’s medium and in the new simplified medium (i.e., medium N°8; DMEM/Ham’s F12 [1:3], 832.2 µM L-glutamine, 0.12 U/mL insulin, 8.3 ng/mL EGF, and 10% FBS) for collagen IV, laminin 1, and K14. The presence of the basement membrane was visible through the positive expression of collagen IV and laminin 1, indicating the preservation of the basement membrane in the DED after de-epidermization, as shown in Figure 5A. There was no difference in the staining between the two media conditions, confirming that the new simplified medium is fully equivalent to the mGreen’s medium for DED constructs seeded with HaCaT cells. Interestingly, there was no K14 staining in the DED constructs maintained in both tested media, as compared to human skin controls (Figure 5D), which showed a strong expression of K14 localized in the basal cells of the epidermis.

### 3.4. Burn Wound Exudate Effects on Keratinocyte Behavior in DED Constructs

**Figure 6:**
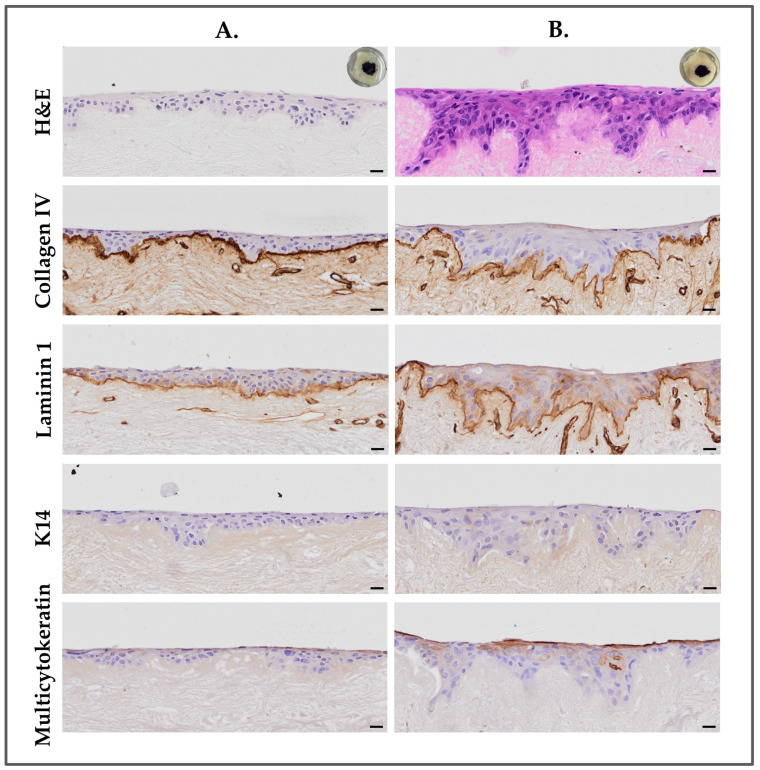
H&E staining and immunohistochemical analysis of collagen IV, laminin 1, and K14 of the DED constructs maintained with different burn wound exudates. (**A**) Early collection exudate group. (**B**) Late collection exudate group. Upper right corners = macroscopic images of tissues stained with MTT. Scale bars = 20 µm. DED, de-epidermized dermis.

Exudates were first tested on primary skin fibroblasts for potential cytotoxicity. The exudates from the early and late collections were used purely at 100% to screen any negative effects on cells. We observed that the burn wound exudates, whether from the early or late collection, promoted cell growth and, therefore, seemed to have positive effects on cells, as shown in Figures S2 and S3. The exudates were then used to replace the air–liquid medium in the DED model. When the DED constructs were maintained in early or late burn wound exudates, the MTT staining results indicated the presence of viable cells, with both conditions showing similar levels of MTT staining intensity, indicating comparable cell viability and proliferation (Figure 6A,B; upper right corner).

The additional information provided by H&E staining and immunohistochemistry further elucidated the interaction between HaCaT cells and DEDs in the presence of the two different exudates (Figure 6). It is of interest to note that DED constructs cultured in the late collected burn wound exudate showed the formation of a deeper epidermis with more invagination of the cells in the dermis than the early collected burn wound exudate. Furthermore, the cells seemed always smaller and pyknotic in the DED constructs maintained in the early burn wound exudate when compared to the late burn wound exudate. This could indicate that the early burn wound exudate could represent an inflammatory status during patient exudation, which induces cell apoptosis. Furthermore, since the late burn wound exudate did not have the same effect on the cells seeded on the DED, it could represent the patient status when the inflammatory state is reduced and permits normal cell adhesion and migration. The same results were observed with the other early and late burn wound exudates tested (Figure S1).

## 4. Discussion

The presented standardization of DED constructs simplifies the procurement and stockage and improves consistency for large batches of substrates, offering a valuable and reproducible tool for comparable experimentation. Herein, we showed that DED can be conserved at 4 °C for up to five years without compromising quality and function. The extended shelf-life under controlled temperature conditions ensures the continuous availability of DED for long-term studies, reducing the costs and efforts associated with frequent sample renewal. With one tissue donation, it is possible to prepare at least three lots of 12 DEDs of 1.5 cm^2^.

Our findings further showed that the composition of the media significantly affects HaCaT cell proliferation and viability on DEDs. Specifically, mGreen’s medium at the air-liquid interface resulted in higher staining intensity and better cellular adhesion compared to the CnT-PR serum-free medium, as confirmed by MTT assay results. Green’s medium is well-established in the literature as the optimal choice for DED air–liquid interface culture methods due to its extensive use and validation in studies focused on keratinocyte culture, differentiation, and stratification [1,5,12,13]. It contains essential components that collectively promote the formation of multilayered, functional epidermal structures [1]. A complete medium comprising DMEM and FBS has also been used extensively to culture HaCaT cells and has demonstrated better results than the CnT-PR serum-free medium for cell seeding in our experiments [29,30]. 

Specifically, we aimed to simplify the composition of Green’s medium for the bottom of the air–liquid interface model. Therefore, different medium combinations were elaborated by reducing specific components without compromising their effectiveness for cell adhesion and migration, which were evaluated. We retained the basal medium containing DMEM and Ham’s F12, which provided amino acids and vitamins, along with FBS, which is crucial for maintaining physiological balance and promoting cellular proliferation [31]. We evaluated the removal of cholera toxin, hydrocortisone, insulin, and EGF. Our results indicated that cholera toxin and hydrocortisone could be omitted without affecting medium functionality on the DED constructs for cell seeding. This was evidenced by the MTT assay, which showed consistent color intensity at the macroscopic level, and by H&E staining, which revealed similar keratinocyte invagination and proliferation regardless of the presence of these components.

Hydrocortisone and cholera toxin have long been used in keratinocyte culture, where they have been described as a stimulator of cell growth [12,32]. The removal of these components could potentially reduce keratinocyte culture confluency, as described in the work of Feisst et al. [17]. This article also mentions a new, effective culture medium in which cholera toxin could be replaced by keratinocyte growth factor (KGF) and a Rho-associated protein kinase (ROCK). However, we utilized HaCaT cells in this study, which do not require these components for proliferation. Furthermore, the purpose set forth herein was to simplify the model. Additionally, DED culture differs from primary keratinocyte culture in monolayer conditions in cell culture flasks. To the best of our knowledge, no studies have previously examined the specific impact of these components on DED culture systems, confirming the novelty of our model.

Insulin and EGF were found to significantly influence outcomes. Samples containing both insulin and EGF exhibited high staining intensity in the MTT assay, and the H&E stains demonstrated increased HaCaT cellular proliferation and invagination compared to those containing neither or only one of these components. Notably, EGF is known to be crucial in re-epithelialization, enhancing keratinocyte proliferation, migration, and formation of a multilayer. It increases the expression of keratinocyte activation markers, demonstrating its critical role in epidermal formation in the DED model [5,33]. Liu et al. [34] demonstrated the importance of insulin in stimulating keratinocyte migration and promoting the formation of epidermal reticular ridges and dermal papillae, although insulin’s effect on keratinocyte migration was found to be independent of EGF presence. Evidence suggests that both components are important for keratinocyte proliferation, either individually or in combination, as also demonstrated by our results.

Our study also explored the role of burn wound exudates on the behavior of keratinocytes on DED constructs. Using exudates collected from burn patients at different stages following burn injury, we demonstrated (i.e., with MTT and H&E staining) differences in their efficacy in promoting keratinocyte adhesion and proliferation. The observed effects could be related to the inflammatory status attributes of burn wound exudates taken from the early stage following a burn injury. To date, few studies have investigated this topic. Only Ono et al. used diluted exudates for keratinocyte culture, demonstrating a positive in vitro effect on cellular proliferation [21]. However, the aforementioned study did not assess differences in burn wound exudation at different times following injury. This effect may be due to the various components present in exudates, including growth factors and cytokines [18,20,21]. One of these growth factors, EGF, plays a crucial role in promoting cutaneous healing through its high capacity for stimulating cellular proliferation, as demonstrated in our previous experiments.

Burn wound exudates harvested at stages close to the burn injury (i.e., early burn wound exudates) or several days later (i.e., late burn wound exudates), when exudation slowly decreases and the wounds “dry out”, were used in the DED air–liquid interface model. In both cases, the results showed keratinocyte attachment to the DED model, as indicated by the MTT assays. More importantly, we were able to determine differences in keratinocyte behavior (i.e., H&E staining), with the late burn wound exudate showing very good keratinocyte adhesion but also significant invagination into the DED dermal layer. This last aspect seemed to be less important with the use of the early burn wound exudate. The late burn wound exudate produced results with HaCaT cells, which were similar to the positive control with mGreen’s medium, although with slightly lower intensity. Interestingly, it was observed that DED constructs maintained in early burn wound exudate presented less or no cellular invagination, with cells remaining only on the DED surface and forming multilayers. The explanation for these distinctions could be attributed to the different physiological stages of wound healing [35,36]. Early burn wound exudate is associated with the initial inflammatory phase, characterized by the recruitment of immune cells to clear debris and prepare the wound bed for healing. During this phase, in vivo, there is limited cellular proliferation and tissue remodeling, as the focus is primarily on inflammation control and debris clearance. In contrast, the late burn wound exudate is produced during the resolution of inflammation, where anti-inflammatory cytokines promote tissue regeneration. The latter resulted in better cellular proliferation and increased invaginations on the DED constructs.

An important point raised in Widgerow’s work [18] is that the inflammatory mediators and cytokines present in burn wound exudate can have toxic and immunosuppressive effects, thereby increasing the patients’ vulnerability to infections and provoking an excessive inflammatory response. These risks often justify early excision of the eschar to prevent such complications. However, our results revealed that cellular proliferation and adhesion are still observed in DEDs maintained with early exudates containing inflammatory components. However, these effects are significantly less pronounced compared to exudates collected at a later phase, where pro-regenerative compounds are more dominant. Overall, the more defined and appropriate window for topically applying cell therapies (e.g., keratinocyte cell sprays) would thus seem to be closer to 4–6 days following trauma to assure full keratinocyte adhesion and function with migration.

The immunohistochemical analysis of laminin 1 and collagen IV, essential components of the basement membrane, on the DED after decellularization confirmed that the basement membrane remained intact. The preservation of these elements promoted keratinocyte adhesion to the dermis, even in the absence of fibroblasts, as highlighted in the work of Deshpande et al. [37] and El Ghalbzouri et al. [27]. This evokes evidence that the DED is particularly effective for tissue culture experiments, providing an ideal ex vivo environment to study keratinocyte adhesion and proliferation. The presence of these two proteins in DED with all tested bottom media, including the new simplified medium N°8, mGreen’s medium, and the two types of exudate, confirmed the good preservation of the basement membrane. This, in turn, facilitated strong cellular adhesion within the matrix.

As revealed by positive control samples of human skin, K14 was localized at the basement membrane [26,38]. However, in our experiments, the K14 protein was not present. The lack of detection of these proteins in our DED seeded with keratinocytes could be attributed to the use of HaCaT cells, which, although useful as a cellular model, do not express keratins in the same way as human primary keratinocytes [5,27,38,39]. 

Within our work, we have demonstrated the comparability of a new simplified medium to that of mGreen’s medium and could, therefore, standardize this major component of the DED model. Importantly, the simplification of the medium was designed for the air–liquid interface to then have a comparative to be adapted to the burn wound exudate groups for validation of the DED model as a simplified burn wound model. Recent studies have also shown that the media may be simplified, as previously mentioned. Specific factors, such as cholera toxin, would be of interest to eliminate in a screening model that is being developed for burn wound exudates. That was the primary intention herein for simplification of the DED preparation standardization. Other manners in DED preparation and standardization may have other nutrient needs for cell adhesion and migration, but with the methodology provided herein, it was notably possible to simplify and observe differences when the bottom media was replaced with burn wound exudates.

The evaluation of different stages of burn wound exudation could be performed to further develop the DED model as a functional testing platform for cutaneous cellular therapies and different combinations of physiological skin cell types. Early burn wound exudates showed significant differences in keratinocyte adhesion and migration when compared to late burn wound exudates. This aspect, observed in situ with the burn wound model developed with DEDs and burn wound exudates, could help to evaluate different therapeutic cell topical delivery protocols for burn patients. In detail, the timing of optimal delivery of cells and their combinations for optimal repair of the tegument could be assessed. In addition, further experiments using primary human keratinocytes, combinations of keratinocytes and fibroblasts, and longer culture periods would be of interest to evaluate the presence of K14 to approach what may be seen in normal human skin. Overall, study limitations specifically comprised the use of HaCaT keratinocytes alone as a model of therapeutic cells. Thus, perspectives to this study may comprise the use of primary keratinocyte and fibroblast cell types from patients in our burn center in order to validate the presented DED model with further enhanced clinical relevance.

## 5. Conclusions

The present study successfully standardized and optimized the DED model for evaluating clinical cutaneous cell therapies, highlighting the importance of insulin and EGF in culture media for promoting keratinocyte proliferation/function in this model. Burn wound exudates, particularly from later harvest stages, significantly enhanced keratinocyte growth, adhesion, and migration, demonstrating their active role in burn wound regeneration. The ex vivo DED model, with a simple preparation method and long-term storage capability, proves to be a valuable tool for regenerative medicine and wound healing research, paving the way for future advancements in clinical cell therapies and assisting clinical quality management of patient care. An immortalized keratinocyte cell line was designed herein to simplify the model and to look particularly at the interface of adhesion and the invasiveness potential with simplified media. Secondly, the influence of burn wound exudates could then be assessed for the same mechanism of adhesion and invasiveness. Further studies are currently underway to assess primary keratinocyte and fibroblast combinations from multiple donors due to the high variability of keratinocyte cell culture from burn patients. Therein, the complex interactions of the dermal structure and function of the various cell types will be addressed. Specifically, these future works will investigate primary dermal fibroblast and keratinocyte combinations with a burn wound model, with exudates collected from wounds of different burn degrees and surfaces. Firstly, the validation of the simplified media for the bottom layer of the air–liquid interface model will be performed. Secondly, different ratios of cell sources (i.e., primary keratinocytes and fibroblasts), along with their delivery, will need to be optimized. This will contribute to enhancing the translatability of the results to clinical burn models, as it will integrate the multi-cell type environment, which is necessary for fully functional ECM generation, remodeling, and modulation.

## Figures and Tables

**Table 1:** Listing of the tested media compositions, ranging from all components used in the formulation (i.e., mGreen’s medium: DMEM and Ham’s F12 [3:1], cholera toxin, hydrocortisone, EGF, L-glutamine, insulin, FBS) to those with less complexity (i.e., complete medium minus different constituents represented in 10 formulations). Components present in each formulation variant are represented in gray. DMEM, Dulbecco’s modified Eagle medium; EGF, epidermal growth factor; FBS, fetal bovine serum.

Medium Type	C ^1^	1	2	3	4	5	6	7	8	9	10	G ^2^
Cholera Toxin		X		X		X		X		X		X
Hydrocortisone			X	X		X		X			X	X
Insulin					X	X			X	X	X	X
EGF							X	X	X	X	X	X

^1^ Complete medium. ^2^ mGreen’s medium.

**Table 2:** Antibody details for the immunohistochemistry assays.

Antibody Type	Clone	Species	Supplier	Dilution
Collagen IV	COL-94	Mouse	Sigma C-1926 (Sigma Aldrich, Buchs, Switzerland)	1:1000
Laminin 1	Polyclonal	Rabbit	Sigma L9393 (Sigma Aldrich, Buchs, Switzerland)	1:500
Cytokeratin 14	LL002	Mouse	Abcam ab7800 (Abcam, Cambridge, UK)	1:2000

**Table 3:** Summary of MTT and H&E staining results for the culture media composition screening.

Groups		C	1	2	3	4	5	6	7	8 *	9	10	G
MTT	Color intensity	+	+	+	+	++	++	+++	+++	+++	+++	+++	+++
	Macroscopic Diameter (+ = < 5.1 mm; +++ = > 8.6 mm)	+	+	+	+	+	+	+++	+++	+++	+++	+++	+++
H&E	Epidermal definition	+	+	+	+	++	++	++	++	+++	+++	+++	+++

MTT color intensity estimations by image J (v. 1.52t) were as follows: + is diffuse staining, ++ is medium intensity and +++ strong intensity. MTT macroscopic diameter was graded as follows: + is less than 5.1 mm, ++ is between 5.2 and 8.5 mm, and +++ is more than 8.6 mm. H&E was graded as follows: + represents “rare cells and no invagination”, ++ represents “low cellular layering and low invagination”, and +++ represents “normal skin-like epidermal cell layering and significant invagination”. * Highlighted in red is the proposed simplified medium.

## Data Availability

The data presented in this study are freely available in the article files.

## Supplementary Materials

The following supporting information can be downloaded at https://www.mdpi.com/article/10.3390/bioengineering11121297/s1, Figure S1: H&E staining of DED constructs with different burn wound exudates; Figure S2: Primary cell survival data in the presence of burn wound exudates; Figure S3: Primary cell survival photographic records in the presence of burn wound exudates.

## Abbreviations

CHUV	Centre hospitalier universitaire vaudois
DED	de-epidermized dermis
DMEM	Dulbecco’s modified Eagle medium
ECM	extracellular matrix
EGF	epidermal growth factor
FBS	fetal bovine serum
h	hour
HaCaT	immortalized human keratinocytes
H&E	hematoxylin & eosin
KGF	keratinocyte growth factor
min	minute
MTT	3-(4,5-dimethylthiazol-2-yl) 2,5-diphenyltetrazolium bromide
PBS	phosphate-buffered saline
PBST	PBS-Tween^®^
P/S	penicillin-streptomycin
ROCK	Rho-associated protein kinase
3T3	murine fetal fibroblasts
