# Supplementary material for: Optimization and Standardization of Stable De-Epidermized Dermis (DED) Models for Functional Evaluation of Cutaneous Cell Therapies
Source: Bioengineering (Basel). 2024 Dec 20;11(12):1297. doi: 10.3390/bioengineering11121297 (PMC11727145; doi:10.3390/bioengineering11121297)
Supplement: Supplementary file 1 [file bioengineering-11-01297-s001.zip › Supplementary Materials.pdf]

bioengineering

Supplementary Materials:

Optimization and Standardization of Stable De-Epidermized Dermis (DED) Models for Functional Evaluation of Cutaneous Cell Therapies

*Xi Chen, Corinne Scaletta, Zhifeng Liao, Alexis Laurent, Lee Ann Applegate * and Nathalie Hirt-Burri **

1. Supplementary Figures

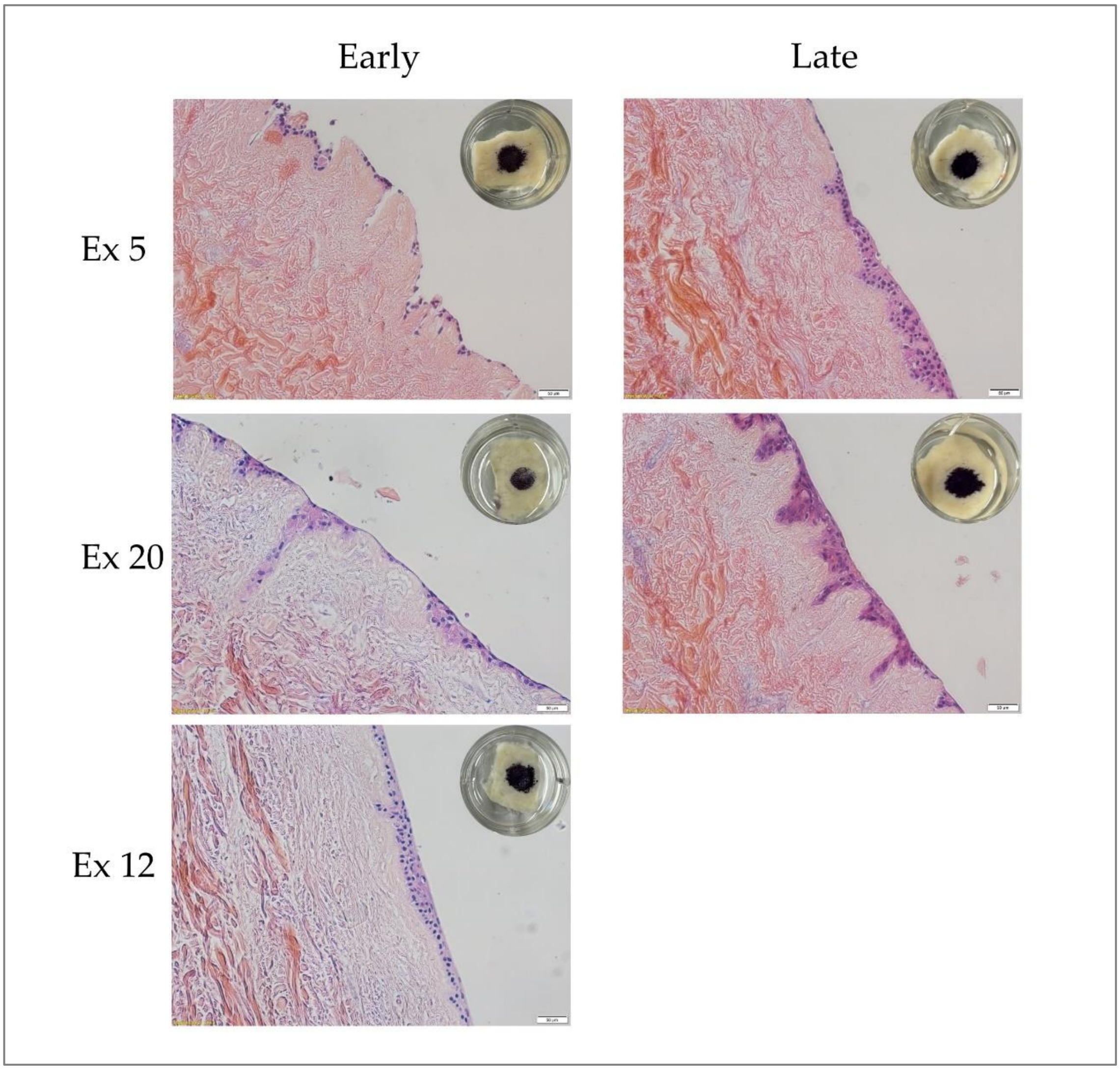

Figure S1. H&E staining of DED constructs with different burn wound exudates. Scale bars = 50 µm. DED, de-epidermized dermis; H&E, hematoxylin & eosin.

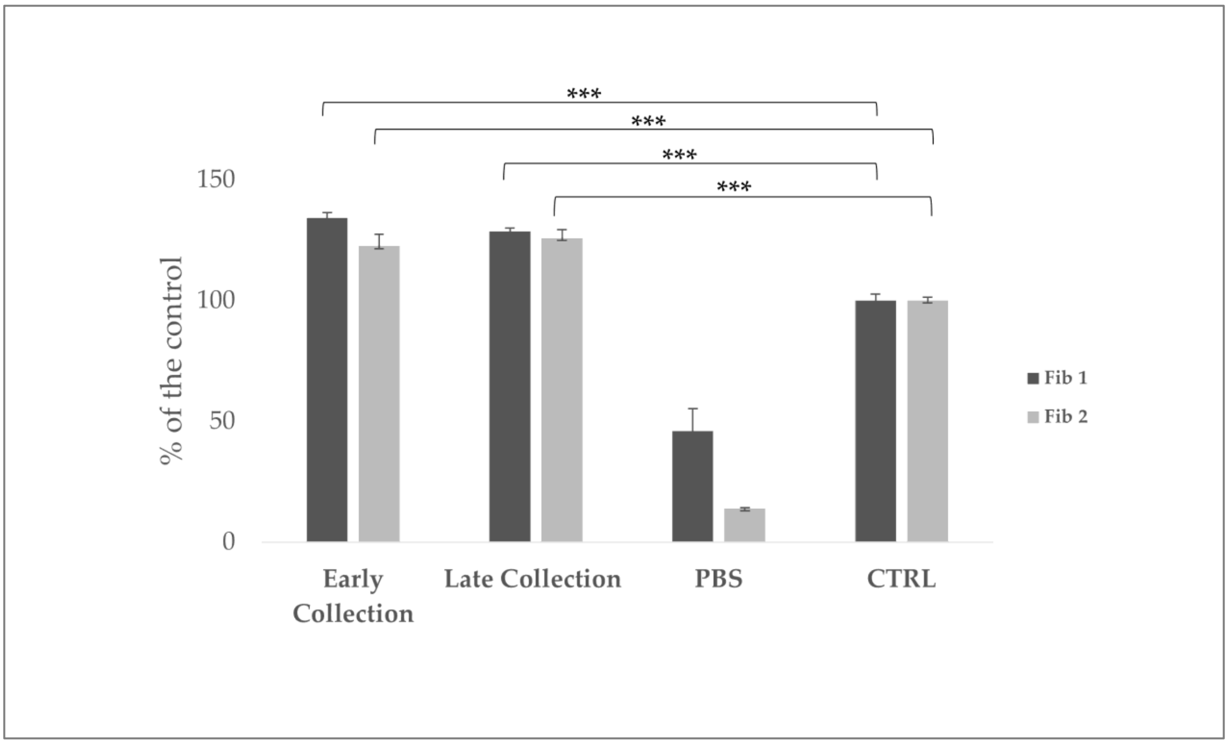

Figure S2. Cell survival percentages relative to control cell treatment with early and late burn wound exudates with two primary fibroblast cell sources. For each group comparative, a Student's t-test was applied and significance with $p < 0.0001$ was indicated by three asterisks ***.

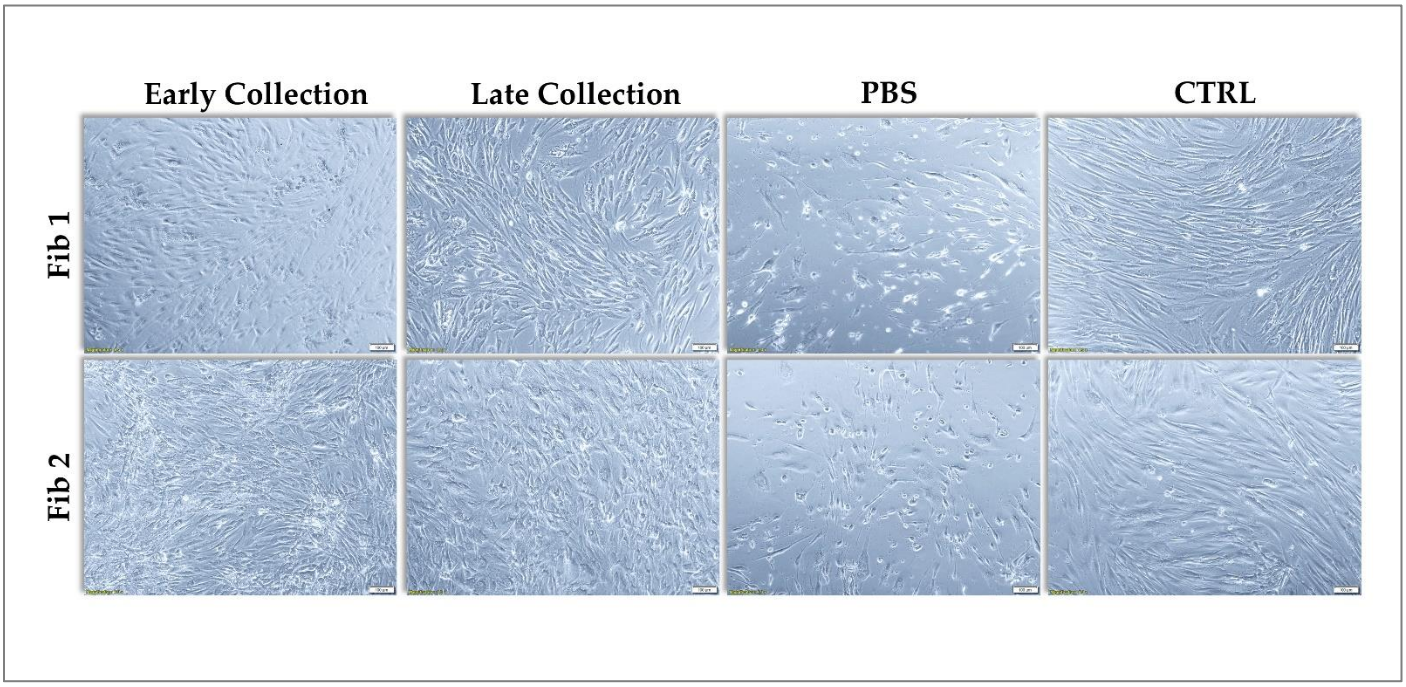

Figure S3. Photographic images of primary fibroblasts 48 h following exposition to early and late burn wound exudates and controls including PBS or culture media alone. Scale bars = 100 μm. PBS, phosphate-buffered saline.
